# Evaluating the impact of a synthetic predator pheromone, a novel pest management tool, on target pests and insect communities in potato fields

**DOI:** 10.1093/jisesa/ieag086

**Published:** 2026-08-19

**Authors:** Laura Martinez, Alayna Trejo, Sabine Paz-Le Draoulec, Gen-Chang Hsu, Jennifer S Thaler

**Affiliations:** Department of Entomology, Cornell University, Ithaca, NY, USA; Department of Entomology, Cornell University, Ithaca, NY, USA; Department of Entomology, Cornell University, Ithaca, NY, USA; Department of Entomology, Cornell University, Ithaca, NY, USA; Department of Entomology, Cornell University, Ithaca, NY, USA; Department of Ecology and Evolution, Cornell University, Ithaca, NY, USA

**Keywords:** chemical ecology, predator cue, non-consumptive effect, foraging behavior, behavioral control

## Abstract

Despite their reputation for species specificity, pheromones can influence interactions across trophic levels. This is particularly relevant in agricultural systems where predator-derived cues could influence pest and natural enemies. The predatory spined soldier bug, *Podisus maculiventris* (Say) (Hemiptera: Pentatomidae), preys upon the Colorado potato beetle, *Leptinotarsa decemlineata* (Say) (Coleoptera: Chrysomelidae), a major pest of solanaceous crops. Previous field work shows that a synthetic aggregation pheromone of *P. maculiventris* reduces beetle larval abundance through non-consumptive effects yet impacts on other herbivores and predators remain unclear. We hypothesized that synthetic predator pheromone cues alter herbivore and predator feeding behavior in an agroecosystem. We evaluated herbivore and natural enemy abundance and diversity in the field, measured predation using sentinel *L. decemlineata* egg clutches, and conducted greenhouse feeding assays with 5 predator taxa. Field results indicated a reduction in *L. decemlineata* larval abundance in pheromone-treated plots without detectable changes in overall herbivore or predator community abundance or diversity. Predation rates in pheromone-treated plots (mean ± SE: 9.8 ± 2.9 eggs consumed per 3 d) were 5 times higher compared to the control (1.4 ± 0.5 eggs consumed per 3 d). Greenhouse assays showed increased egg consumption by *P. maculiventris*. These findings suggest that the synthetic predator pheromone may enhance the foraging behavior of *P. maculiventris*, providing a potential mechanism for reduced *L. decemlineata* populations and a promising tool for potato pest management with minimal non-target impacts.

## Introduction

Semiochemicals, especially sex pheromones, have become essential tools in agricultural pest management, with applications ranging from population monitoring and mass trapping to attract-and-kill, push–pull systems, and mating disruption. Their high specificity and relatively low environmental footprint make them attractive components of integrated pest management (IPM) programs (Sharma et al. 2019, Rizvi et al. 2021). For example, sex pheromones are widely deployed to disrupt mating in orchard pests such as the codling moth (*Cydia pomonella*), where pheromone-based disruption has reduced insecticide inputs and becomes a cornerstone of apple and pear IPM (Light et al. 2001, Witzgall et al. 2008). Along with sex pheromones, aggregation pheromones represent a promising new approach to manipulate pest populations because they attract and retain both sexes. A well-known example is the synthetic aggregation pheromone of the boll weevil (*Anthonomus grandis*), which has been widely used for monitoring and mass trapping in cotton production (Leonhardt et al. 1988). Similarly, aggregation pheromones have been successfully applied in other agricultural systems. An aggregation pheromone blend common across thrips has been used to manage greenhouse and field pests, including western flower thrips (*Frankliniella occidentalis*), through attract-and-kill strategies and behavioral interference (Kim et al. 2023). Another notable example that works in field settings is vittatalactone, an aggregation pheromone that successfully attracts both male and female striped cucumber beetles (*Acalymma vittatum*) (Weber et al. 2023).

However, the long-held assumption that pheromones act with strict species specificity is increasingly challenged with abundant evidence that pheromones influence interactions across multiple trophic levels. For instance, the bark beetle aggregation pheromone, ipsdienol, acts not only as an intraspecific attractant but also as a kairomone for its predator, the checkered beetle (*Thanasimus dubius*) demonstrating how pheromone signals can be co-opted across trophic levels (Dahlsten et al. 2004). In addition, unlike sex pheromones, many aggregation pheromones are multicomponent blends produced and used by multiple taxa. These blends often include plant-derived volatiles or structurally similar convergent compounds, making them more likely to influence non-target organisms. For example, in the squash (*Cucurbita pepo*) system, squash bug (*Anasa tristis*) has been shown to eavesdrop on vittatalactone, the male-produced aggregation pheromone of the co-occurring striped cucumber beetle (*A. vittatum*) and increase its oviposition (Brzozowski et al. 2022). In another example, non-host green leaf volatiles (GLVs) from deciduous trees can also inhibit bark beetle pheromone responses, discouraging colonization of unsuitable hosts (Huber et al. 2021). Collectively, these examples demonstrate that semiochemicals often function through the community, influencing herbivores, predators, and parasitoids. As agroecosystems contain multiple interacting taxa, assessing these cross-level effects is essential for the ecologically safe and effective deployment of pheromone-based tools.

Pheromones and predator-associated volatiles can elicit strong non-consumptive effects (NCEs) on prey as individuals alter behavior to avoid predation. Exposure to predator odors can trigger dispersal, reduce feeding, and deter oviposition, ultimately suppressing pest populations. For example, aphids detect and avoid surfaces contaminated with chemical cues from lady beetles (Ninkovic et al. 2013) and reduce phloem ingestion in the presence of a lady beetle volatile cue (Kansman et al. 2023). Such behavioral shifts represent an underexplored yet promising avenue for pest suppression, where volatiles can deter pests and suppress feeding, leading to yield improvements (Martinez et al. 2025). In addition to directly influencing herbivore behavior, predator-associated cues can induce physiological and defensive responses in plants, which may further reduce herbivore performance through plant-mediated mechanisms. However, as with other semiochemicals, predator cues may also affect non-target species. Predator-derived volatiles are likely to influence a wide array of arthropods, including beneficial predators and alternative prey, thereby reshaping community interactions. For example, Wen et al. (2023) found that a rove beetle pheromone spray reduced predatory spiders while overall arthropod diversity remained stable, indicating limited off-target effects. Still, many predatory insects respond to volatile cues that overlap chemically with herbivore-induced plant volatiles (HIPVs), which are broadly detectable by multiple organisms and can generate non-target and community-level effects (Kaplan 2012).

This study focuses on 1 such system involving the spined soldier bug, *Podisus maculiventris* (Say) (Hemiptera: Pentatomidae), a generalist predator and key natural enemy of the Colorado potato beetle (*Leptinotarsa decemlineata*) (Say) (Coleoptera: Chrysomelidae). Colorado potato beetle is among the most destructive insect pests of potato worldwide because of its capacity for rapid defoliation and widespread insecticide resistance (Alyokhin et al. 2022). Male *P. maculiventris* produces an aggregation pheromone that attracts both sexes and can modify prey responses, including reducing leaf consumption and larval abundance (Hermann and Thaler 2014, Aflitto and Thaler 2020). There are several hypotheses for why this pheromone may affect the community of herbivores or natural enemies in the system. Because this cue aggregates conspecifics, it may intensify intraspecific competition among naturally occurring or augmented *P. maculiventris* when prey are limited or alter intraguild interactions. *P. maculiventris* is known to act as an intraguild predator on the juveniles of other soft-bodied predators like lady beetles (Hough-Goldstein et al. 1996, Mallampalli et al. 2002). Conversely, herbivores may interpret the same cue as evidence of increased predation risk and adjust their behavior by reducing feeding, avoiding treated areas, or altering oviposition to lower the likelihood of predator encounter (Werner and Peacor 2003, Hermann and Thaler 2014). The synthetic pheromone blend consists of α-terpineol, terpinen-4-ol, benzyl alcohol, linalool, and (*E*)-2-hexenal (Aldrich et al. 1984). Two components, linalool and (*E*)-2-hexenal, are also GLVs released by potato plants following herbivore injury, and HIPVs are broadly used by insects across taxa as indicators of herbivore activity, plant damage, and changes in the local risk landscape (Heil 2014). This chemical overlap between predator cues and plant volatiles suggests potential for community-level, non-target effects when the synthetic blend is deployed in the field.

In this study, we evaluate how a synthetic blend mimicking *P. maculiventris* aggregation pheromone influences the target pest, the Colorado potato beetle and the associated arthropod communities as well as enemy–pest interactions in potato fields. We hypothesized that the deployment of the predator pheromone would reduce Colorado potato beetle abundance, alter herbivore and predator communities, increase predation on Colorado potato beetle eggs, and influence herbivore and predator feeding behavior to *Podisus-*specific cues, either directly or indirectly through pheromone-induced changes in the abundance or behavior of naturally occurring predators. We used field experiments to test the effects of the synthetic predator pheromone on herbivore and natural enemy abundance and diversity using 2ly D-vac sampling and visual counts. We tested the effects of pheromone on predation rates by deploying sentinel beetle egg clutches across the potato growing season. We conducted complementary greenhouse assays to test whether the pheromone affected predation rates of beetle eggs by dominant field predators. Understanding these dynamics is crucial for assessing the non-target and community-level impacts of deploying predator pheromones as a novel biocontrol tool in agroecosystems.

## Methods

### Field Experiments

#### Field Set-up

On 3 May 2024, *Solanum tuberosum* (cv. Yukon Gold) potatoes were planted at Homer C. Thompson Vegetable Research Farm in Freeville, New York (42.517895, −76.332011). The variety Yukon Gold was selected due to its extensive use in prior research in this system (Hermann and Thaler 2014, Aflitto and Thaler 2020). Before planting, the field was fertilized with Triple 13 NPK at a rate of 1,289 kg/ha. The experimental layout consisted of 36 single rows of potatoes, each row measuring 30 m in length. Potato seeds were planted 0.3 m apart within rows, with a row spacing of 0.9 m. The field was organized into 3 blocks (A, B, and C), each containing 12 parallel rows. Blocks were separated by approximately 4 m of bare soil and were oriented from right (A) to center (B) to left (C) of the site.

#### Predator Pheromone Release

The synthetic predator pheromone used in this study mimics the male aggregation pheromone of *P. maculiventris*, which is naturally released in spring to attract females. The dispenser containing the predator pheromone was made from a modified opaque brown 2-ml Eppendorf tube with three 0.55-mm holes in the top. Each dispenser was filled with 594.1 µl of an in-house blend of a synthetic male *P. maculiventris* dorsal abdominal gland pheromone mixture (354.0 µl (E)-2-hexenal, 216.0 µl alpha terpineol, 2.4 µl linalool, 2.0 µl terpinen-4-ol, and 19.7 µl benzyl alcohol) (Aldrich et al. 1984) and affixed to a highlighter green marking flag 20 cm high. Control plots received an empty tube attached to a marking flag. Pheromone dispensers have been shown to exert NCEs within approximately 1 m of their placement (Aflitto and Thaler 2020, Martinez et al. 2025). To account for this, plots were designed to be 2 m long and included 2 pheromone dispensers per plot. The dispensers emitted the pheromone at an average rate of 0.23 mg/h (Aflitto and Thaler 2020). This release rate was selected based on dispenser characterization described by Aflitto and Thaler (2020). Although individual *P. maculiventris* have been reported to produce up to approximately 1 mg of aggregation pheromone (Aldrich et al. 1984), natural pheromone exposure experienced by Colorado potato beetles in the field likely varies with predator density, spatial distribution, and environmental conditions. Consequently, our dispenser was intended to provide a biologically relevant pheromone stimulus rather than to replicate natural field concentrations exactly.

Pheromone treatments were deployed on 30 May 2024 to coincide with the emergence of overwintering Colorado potato beetle adults. Within each row, plots consisted of 6 plants spaced 30 cm apart in a single row, with 2 pheromone flags placed adjacent to the second and fourth plants. A 5-m buffer of untreated potato plants was maintained between plots. This spacing was selected based on previous field experiments in our lab indicating no effect on beetle abundance or feeding beyond approximately 1 m from the pheromone dispensers and was considered sufficient to minimize potential pheromone movement among adjacent plots (Aflitto and Thaler 2020, Martinez et al. 2025). To further reduce side-by-side overlap of treatments, plots were staggered between rows.

Each row contained 2 pheromone-treated plots and 2 control plots. Pheromone-treated plots received 2 open-dispersion volatile dispensers, while control plots received 2 empty dispensers. Each plot type included 2 distinct sampling methods, as described below; and treatments were randomized across all rows for a total of 144 plots. Dispensers were left in the field throughout the measurement period and were replaced every 1.5 wk.

#### Field Sampling

We measured the effect of predator pheromone on the abundance of potato herbivores and natural enemies using 2 sampling methods. Starting 4 June 2024, and continuing weekly for 7 wk, we conducted visual counts of Colorado potato beetle predators, the Colorado potato beetle (eggs, larvae, and adults), and other potato pests present on the 6 plants within each of the 72 plots (half control, half with pheromone). Colorado potato beetle predators were identified based on published literature describing established natural enemies of the Colorado potato beetle (Weber et al. 2022). Insects were identified visually in the field and were not collected during visual surveys. For each plant, the observers positioned themselves approximately 15 to 30 cm from the plant material and conducted a systematic inspection. Observers gently manipulated the stems and foliage to examine both the upper and lower surfaces of all leaves, including those near the base of the plant, to ensure full coverage of the plant canopy. To minimize potential observer bias, observers were rotated among plots during each sampling period so that no observer consistently sampled the same plots. In addition, all observers had prior entomological experience and received additional training before sampling on the identification of arthropod taxa commonly encountered in potato fields. An identification guide was available during each sampling event, and a trained entomologist was available to verify uncertain field identifications.

In addition to weekly visual estimates, we used a handheld D-vac device (Model 122 Rincon-Vitova Insectaries, Ventura, California, United States) on the other 72 plots (half control, half with pheromone). Each plant was vacuumed for 5 to 10 s. Samples from all 6 plants were placed into a collection bag labeled with block, plot ID, row, date of collection and placed in the freezer.

#### Insect Identification

All observed organisms were identified to family, with taxa of particular importance for Colorado potato beetle predation and potato pest status identified to finer taxonomic resolution when possible. Focal herbivores included the potato leafhopper, *Empoasca fabae* (Harris) (Hemiptera: Cicadellidae); the Colorado potato beetle, *Leptinotarsa decemlineata* (Say) (Coleoptera: Chrysomelidae); aphids (Hemiptera: Aphididae); the tarnished plant bug, *Lygus lineolaris* (Palisot de Beauvois) (Hemiptera: Miridae); and flea beetles (Coleoptera: Chrysomelidae: Galerucinae) (Weber et al. 2022). Predators were identified to family and included lady beetles (Coleoptera: Coccinellidae), minute pirate bugs (Hemiptera: Anthocoridae), spiders (Araneae: Araneidae), damsel bugs (Hemiptera: Nabidae), and lacewings (Neuroptera: Chrysopidae, Hemerobiidae). Species-level identification was conducted for key predators of the Colorado potato beetle, including *Lebia grandis* (Coleoptera: Carabidae) (Hentz) and *Podisus maculiventris* (Hemiptera: Pentatomidae) (Say). Lady beetles were further identified to *Coleomegilla maculata* (Coleoptera: Coccinellidae) (De Geer), a native, naturally occurring predator of Colorado potato beetle, whereas other coccinellids were retained at the family level for community analysis. Herbivore and predator taxa representing less than 1% of total observations were not identified further, as these uncommon taxa were unlikely to play a substantial role in pest suppression or plant damage within this system (Chang and Snyder 2004). Although excluding rare taxa may slightly underestimate richness and diversity, these taxa occurred at very low frequencies and were not expected to substantially influence community-level patterns or conclusions.

#### Egg Predation Rates in the Field

To quantify Colorado potato beetle egg predation rates in the field over the potato growing season, we deployed sentinel beetle eggs weekly from 7 June to 19 July 2024. We prepared sentinel eggs using 3-wk-old potted potato plants grown in the greenhouse and fresh egg clutches (20 to 40 eggs per clutch) collected from a lab colony of the Colorado potato beetle. We used a paired design where an egg clutch was glued to the lower side of a leaf (the “predator-exposed” clutch) and another clutch was glued to the lower side of a nearby leaf of the same plant enclosed in a mesh bag (the “control” clutch). Each week, we randomly selected 5 to 7 control and pheromone-treated plots across our study site and deployed one plant with sentinel eggs in each plot for 3 d (no repeated deployments in the same locations over the season). The number of lost eggs in each clutch was determined as the difference between the initial number of eggs and the number of remaining intact eggs (partially damaged and shrunk eggs were counted as lost eggs as they were not able to hatch). To account for non-predation egg mortality, background egg loss from the paired control clutch was scaled to the size of the predator-exposed clutch and subtracted from observed egg loss to estimate predation. The average non-predation mortality was low and similar in the control and pheromone-treated plots (mean ± SE; control plot: 0.77 ± 0.23 eggs per 3 d; pheromone-treated plot: 0.75 ± 0.27 eggs per 3 d). Observations with clutches (either control or predator-exposed) that were missing or hatched were excluded, giving a final sample size of 50 (30 in the control plots vs. 20 in the pheromone-treated plots) in the analysis.

### Lab Experiments

#### Predation Assays

Greenhouse feeding assays were conducted using the dominant Colorado potato beetle egg predators that were also abundant in the field, focusing on the species that could be reliably collected or maintained under laboratory conditions. These included *Coleomegilla maculata* adults and fourth-instar larvae, *Lebia grandis* adults, *Chrysoperla rufilabris* third-instar larvae, and *P. maculiventris* adults. The assays took place in the greenhouse facility in Upstate, New York. Minute pirate bug *Orius insidiosus* was also assessed through this study due to its high abundance in the field; however, there was no clear consumption evidence of eggs, and no further analysis was performed. *Coleomegilla maculata* adults, *Lebia grandis* and *P. maculiventris* adults were field collected from experimental plots located in Freeville, New York in August 2024. *Chrysoperla rufilabris and Orius insidiosus* were purchased from ARBICO Organics, Oro Valley, Arizona. Additional adult *Coleomegilla maculata* and all fourth-instar *Coleomegilla maculata* larvae were taken from our lab-colony reared in a growth chamber maintained on a 16:8 L:D cycle and fed on pea aphids.

For the greenhouse assays, predators were randomly selected from the lab colony once predators started reproducing or assays were done following field collection. Prior to the experiment, predators were isolated individually without food for 24 h to standardize hunger levels.

Each replicate contained a single potato plant with a set number of *Leptinotarsa decemlineata* eggs affixed to the abaxial side of a single, mid-height leaf blade. The beetle eggs were collected from lab-maintained colonies in a growth chamber on a 16:8 L:D cycle. Temperature was maintained at approximately 28.2 °C. All eggs were collected within 24 h before the start of the assays. Plants were spaced equally in the greenhouse, about 0.5 m apart. Individual predators were introduced to the plants randomly assigned to either the control or the treatment group. Plants in the control group were provided rubber septas charged with 250 μl of hexane solution and soaked overnight. Plants in the treatment group had rubber septas charged with 250 μl of 1% pheromone solution (see above) diluted with hexane, attached to a leaf around 10 to 15 cm across from the egg-affixed leaf. All plants were covered by perforated bread bags and secured at the base of the plant with a rubber band. Replicates were evenly divided between control and pheromone treatments. Due to space constraints, the experiments were carried out over sequential trials, each containing an equal number of control and pheromone replicates. Total eggs consumed were counted after 24 or 48 h. Predators that died or pupated during the assay were omitted from analysis.

The predation assays were done with *C. maculata* adults (*n* = 128; 63 control, 65 pheromone), *C. maculata* fourth-instar larvae (*n* = 27; 15 control and 12 pheromone), *L. grandis* adults (*n* = 32; 16 control and 16 pheromone), *C. rufilabris* third-instar larvae (*n* = 46; 23 control and 23 pheromone), and *P. maculiventris* adults (*n* = 62; 31 control and 31 pheromone; this assay was conducted for 48 h).

### Data Analysis

#### Abundance of the Colorado Potato Beetle

We analyzed the abundance of the Colorado potato beetle (*L. decemlineata*) separately for the following biological and statistical reasons: (i) It is the focal pest species in this system, (ii) it has known behavioral responses to predator-derived cues (Helms et al. 2019, Aflitto and Thaler 2020), (iii) it was not found in D-vac samples, and (iv) its statistical distribution differed from that of other herbivores in the system. We fit a generalized linear mixed-effects model with beetle larval abundance as the response variable, pheromone treatment as the fixed effect, with week and plot nested within the row and block as the random effects.

#### Abundance, Richness, and Diversity of Arthropod Communities

To evaluate how pheromone treatment influenced herbivore and predator community structure, we calculated total abundance, mean abundance per taxonomic group, richness, and Shannon diversity for each field sample. Herbivore and predator datasets were first filtered to include only non-predatory taxa or predatory taxa only, and total counts were summed within each combination of block, row, plot, treatment, sampling method, week, and taxonomic group. Community metrics were calculated at the level of taxonomic groups (family-level units), including taxonomic richness (number of families detected), mean abundance per family, and Shannon diversity using the “vegan” package (Oksanen et al. 2024). Although community metrics were calculated at the family level, which may mask species-specific responses within some groups, potato arthropod communities are typically dominated by a relatively small number of herbivore and predator taxa. Consequently, we expect family-level metrics to capture the dominant community responses to pheromone deployment, while recognizing that species-level responses within families may have gone undetected.

Abundance was log-transformed to meet model assumptions and analyzed with a linear mixed-effects model including treatment as the fixed effect and week and plot nested within block as the random effects. Richness was modeled using a generalized linear mixed model with a Poisson error distribution, with treatment included as the fixed effect and week and plot nested within block as the random effects. This model was fitted via the glmmTMB() function in the R “glmmTMB” package (Brooks et al. 2017). The effects of treatment on Shannon diversity were evaluated using a linear mixed-effects model with treatment as the fixed effect and week and plot nested within block as the random effects. We checked model assumptions using the simulated scaled residuals generated from the simulateResiduals() function in the R “DHARMa” package (Hartig 2022).

#### Abundance of Selected Predators and Herbivores

To evaluate the effects of pheromone treatment on herbivorous and predator arthropod abundance across taxonomic groups, we constructed generalized linear mixed models using the lme4 package (Bates et al. 2015). Herbivore and predator datasets were filtered to include taxa that were consistently detected across sampling events. The selected herbivores included *Cicadellidae* (potato leaf hopper)*, Galerucinae* (flea beetles)*, Miridae* (tarnished plant bug), and *Aphididae* (Aphids); the selected predators included *P. maculiventris*, Neuroptera (lacewings), Coccinellidae (lady beetles), Nabidae (damsel bugs), Araneidae (spiders), and Anthrocoridae (minute pirate bugs). Abundance for each taxonomic group was calculated as the mean number of individuals collected within each combination of block, plot, row, treatment, sampling method, and week.

We modeled herbivore abundance as a function of treatment, taxonomic group, and their interaction using a negative binomial generalized linear mixed model (GLMM). The full model included the interaction between treatment and taxonomic group as fixed effects, with plot nested within block and week included as random effects. A negative binomial error distribution was used to account for overdispersion in count data. Post hoc comparisons among treatment levels within each taxonomic group were performed using pairwise contrasts of estimated marginal means (EMMs) with the emmeans package (Lenth and Piaskowski 2025).

#### Egg Predation in the Field

To compare the egg predation rates between the control and the pheromone-treated plots, we fit a generalized linear model with the number of predated eggs as the response, and plot treatment type and week as the predictors. We used a negative binomial distribution with a log link function to account for data overdispersion. The model was fitted via the glmmtmb() function in the R “glmmTMB” package (Brooks et al. 2017). We checked model assumptions using the simulated scaled residuals generated from the simulateResiduals() function in the R “DHARMa” package (Hartig 2022). We used the Wald chi-square test to assess predictor significance using the Anova() function (type II sums of squares) in the R “car” package (Fox and Weisberg 2019). The analysis was performed in R version 4.3.3 (R Core Team 2025).

#### Egg Predation in the Greenhouse

We analyzed the probability of egg consumption by 4 predator species (*P. maculiventris, C. maculata*, *L. grandis*, and *C. rufilabris*) using a generalized linear mixed model fitted with the glmmTMB package (Brooks et al. 2017). The response variable was the number of eggs consumed relative to the number available at the start of the assay, modeled as a binomial response. Fixed effects included treatment, predator life stage/species, their interaction, and assay length as a covariate. Random effects included trial identity since experiments were conducted sequentially, and plant identity. The species *C. maculata* was separated to analyze the treatment effect on egg consumption in larvae and adult stages. *Coleomegilla maculata* was selected because it represented the highest proportion of lady beetles observed in the field, while *L. grandis* was included due to its role as an important specialist predator of the Colorado potato beetles. All analysis was conducted in R version 4.4.0 (R Core Team 2025).

## Results

### Herbivore Abundance and Community Structure

**Fig. 1.**
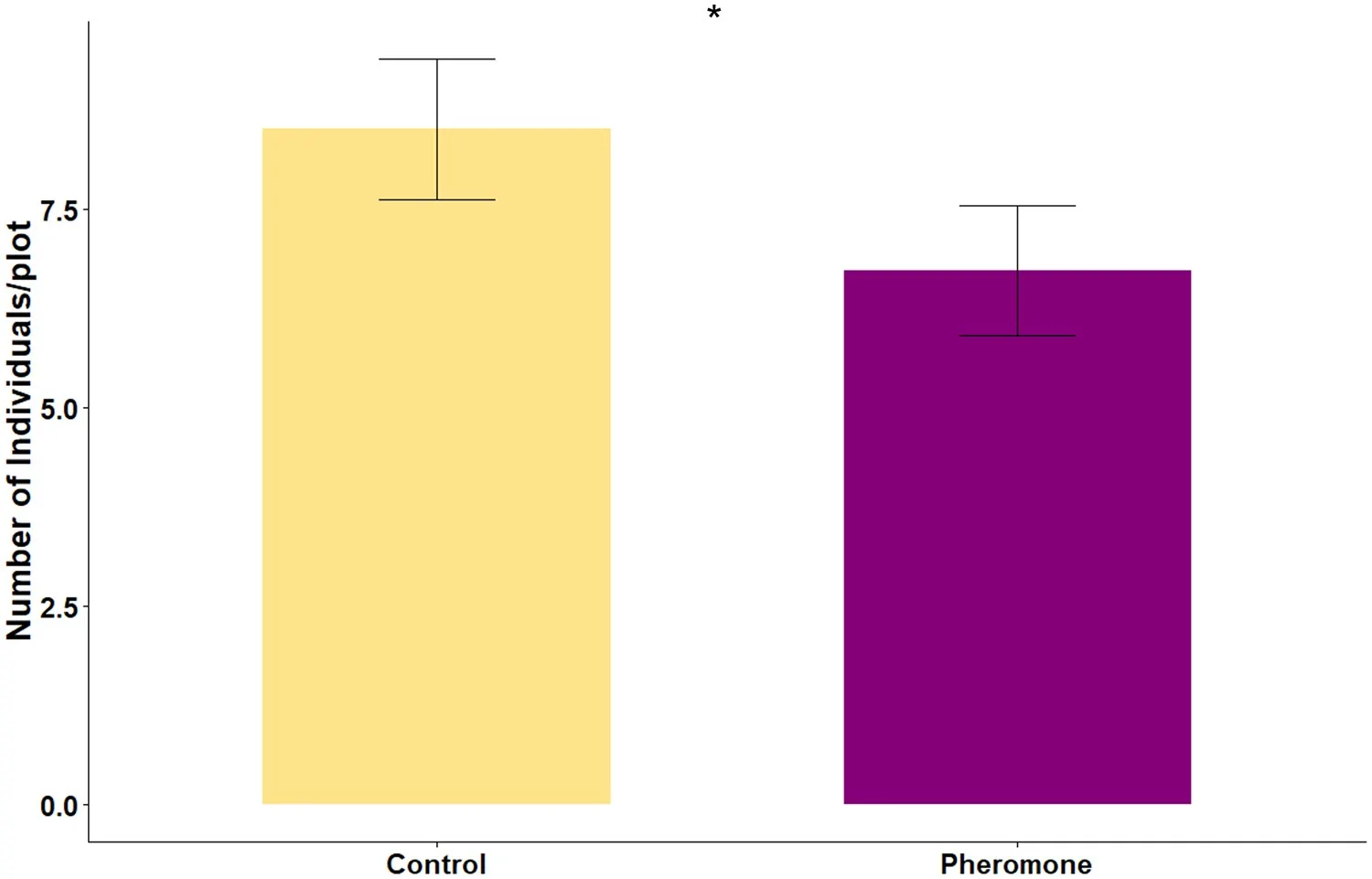
Mean (± SE) number of Colorado potato beetle larvae per plot in control and pheromone-treated plots averaged across the sampling period. Raw observed means were 8.51 ± 0.89 larvae per plot in the control treatment (*n *= 177 observations) and 6.73 ± 0.82 larvae per plot in the pheromone treatment (*n *= 159 observations). Error bars represent ±1 SE. Asterisk indicates a significant treatment effect (*P *< 0.05).

While the abundance of the target herbivore, the Colorado potato beetle, was reduced by the pheromone, none of the other herbivores were affected. The estimated mean abundance of Colorado potato beetle larvae was 32% lower in pheromone-treated plots than in control plots (5.53 vs. 8.08 larvae per plot; *z *= −2.51, *P *= 0.01; Fig. 1).

The presence of the synthetic predator pheromone did not significantly alter herbivore abundance, richness, or diversity of the rest of the herbivore community over the 7-wk sampling period (Supplementary Fig. S1 and Supplementary  Table S1). The herbivore community was dominated by potato leafhoppers, which comprised approximately 79% of all herbivores observed, followed by the Colorado potato beetles (6%), aphids (5%), tarnished plant bugs (5%), and flea beetles (4%). Together, these taxa accounted for approximately 99% of the herbivore community.

**Fig. 2.**
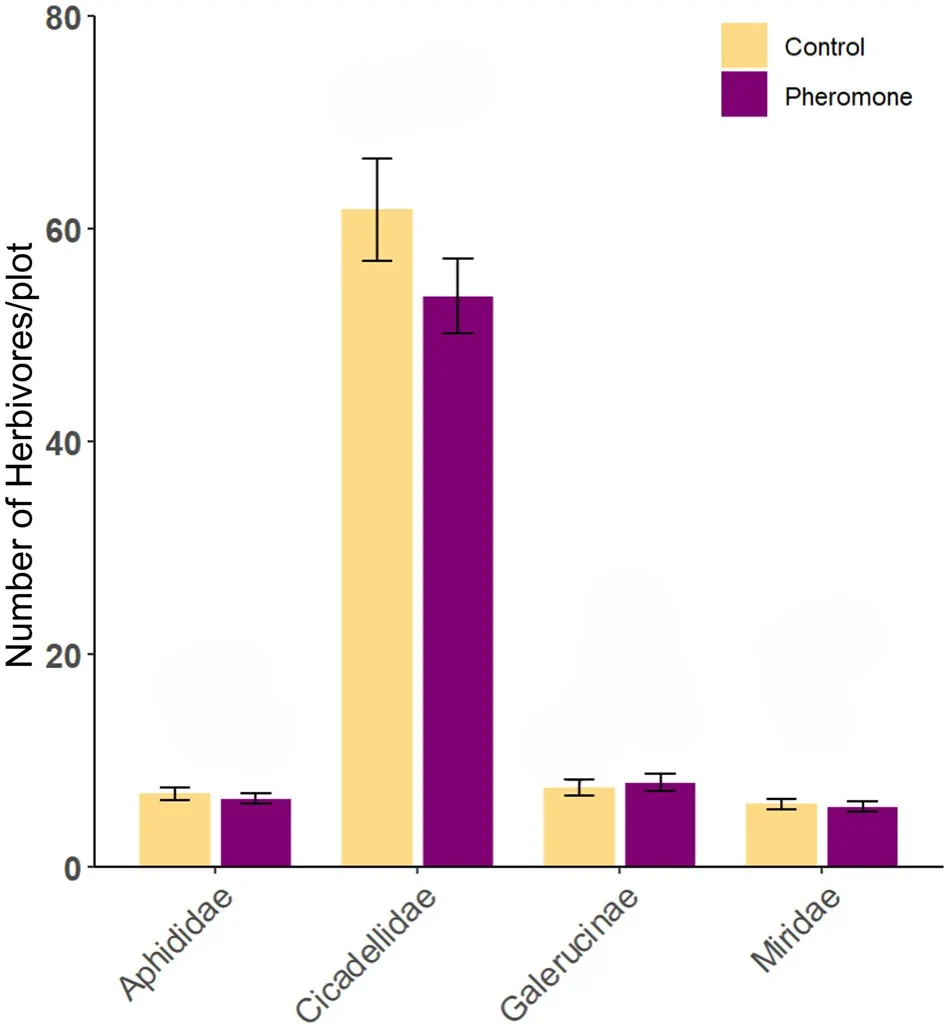
Average herbivore number per plot across 2 months in control and pheromone-treated plots. Herbivores are shown by family and were the major herbivore families found in the field. Aphididae (aphids), Cicadellidae (potato leafhopper), Galerucinae (flea beetles), and Miridae (tarnished plant bugs). Error bars represent standard errors.

Aphids, potato leafhoppers, flea beetles, and tarnished plant bugs did not differ significantly in abundance between the control and pheromone treatments (Fig. 2; Table 1).

**Table 1.** Control/pheromone abundance ratios (± SE) for dominant herbivore taxa. Ratios were estimated from generalized linear mixed models; values significantly different from 1 would indicate treatment effects. No significant differences were detected (*P *< 0.05)

Taxonomic group	Control/Pheromone ratio	SE	*Z*	*P*-value
***Aphididae***	1.10	0.09	1.09	0.28
***Cicadellidae***	1.07	0.07	1.04	0.29
***Galerucinae***	0.93	0.09	−0.72	0.47
***Miridae***	1.10	0.09	0.14	0.89

### Predator Community Structure

Exposure to the synthetic predator pheromone did not significantly alter predator abundance, richness, or diversity over the 7-wk sampling period (Supplementary Fig. S2 and Supplementary  Table S2). The predator community was dominated by lady beetles, which comprised approximately 48% of all predators observed, followed by minute pirate bugs (22%), spiders (12%), damsel bugs (6%), spined soldier bugs (5%), and lacewings (4%). Together, these taxa accounted for approximately 97% of the predator community.

**Fig. 3.**
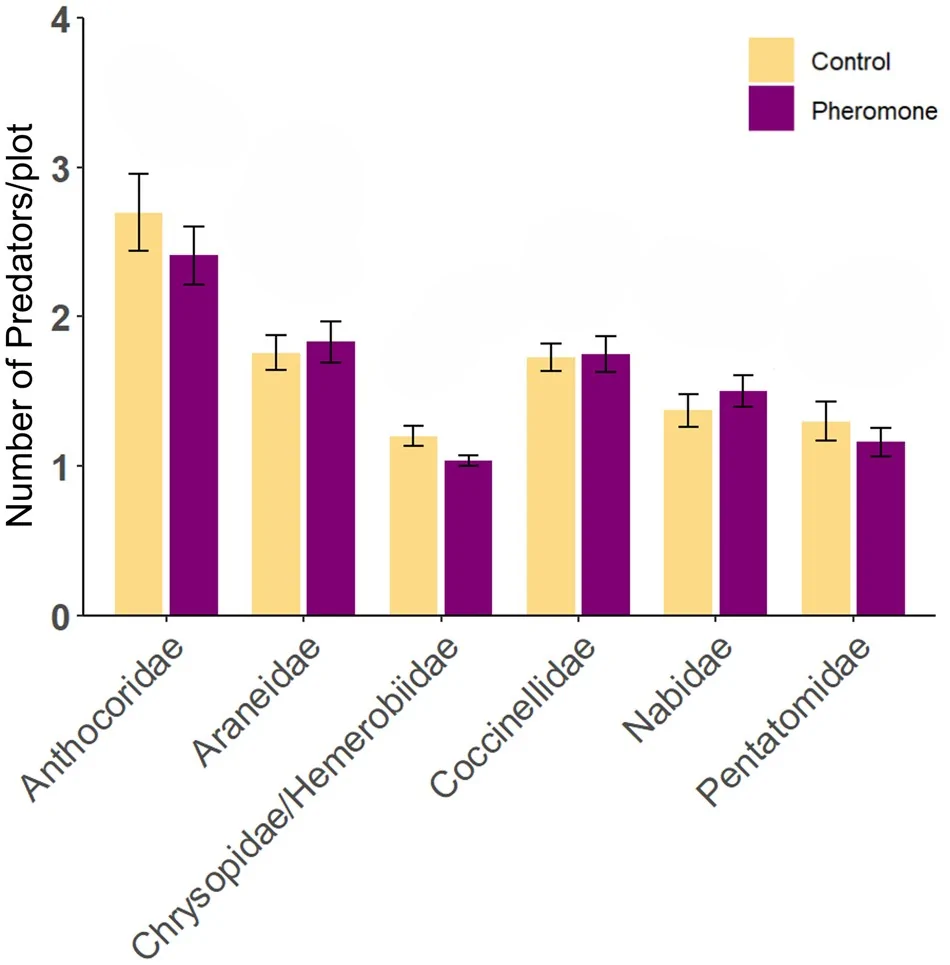
Average predator number per plot across 2 months in control and pheromone treated plots. Predators are shown by family and are the most abundant in the field, Anthocoridae, Araneidae, Neuroptera (Chyrsopidae/Hemerobiidae), Coccinellidae, Nabidae, and Pentatomidae. Error bars represent standard errors.

The most abundant predators in the potato field-minute pirate bugs, spiders, lady beetles, damsel bugs, lacewings, and spined soldier bugs, did not differ significantly in abundance between the control and pheromone treatments (Fig. 3; Table 2).

**Table 2.** Control/pheromone abundance ratios (± SE) for dominant predator taxa. Ratios were estimated from generalized linear mixed models; values significantly different from 1 would indicate treatment effects. No significant differences were detected (*P *< 0.05)

Taxonomic group	Control/Pheromone ratio	SE	*Z*	*P*-value
***Anthocoridae***	1.11	0.17	0.69	0.49
***Araneidae***	0.81	0.25	−0.68	0.50
***Coccinellidae***	0.99	0.09	−0.14	0.89
***Nabidae***	0.92	0.24	−0.33	0.74
***Neuroptera***	1.12	0.35	0.46	0.65
***Podisus***	1.11	0.38	0.36	0.76

### Predation Rates of Colorado Potato Beetle Eggs in the Field

**Fig. 4.**
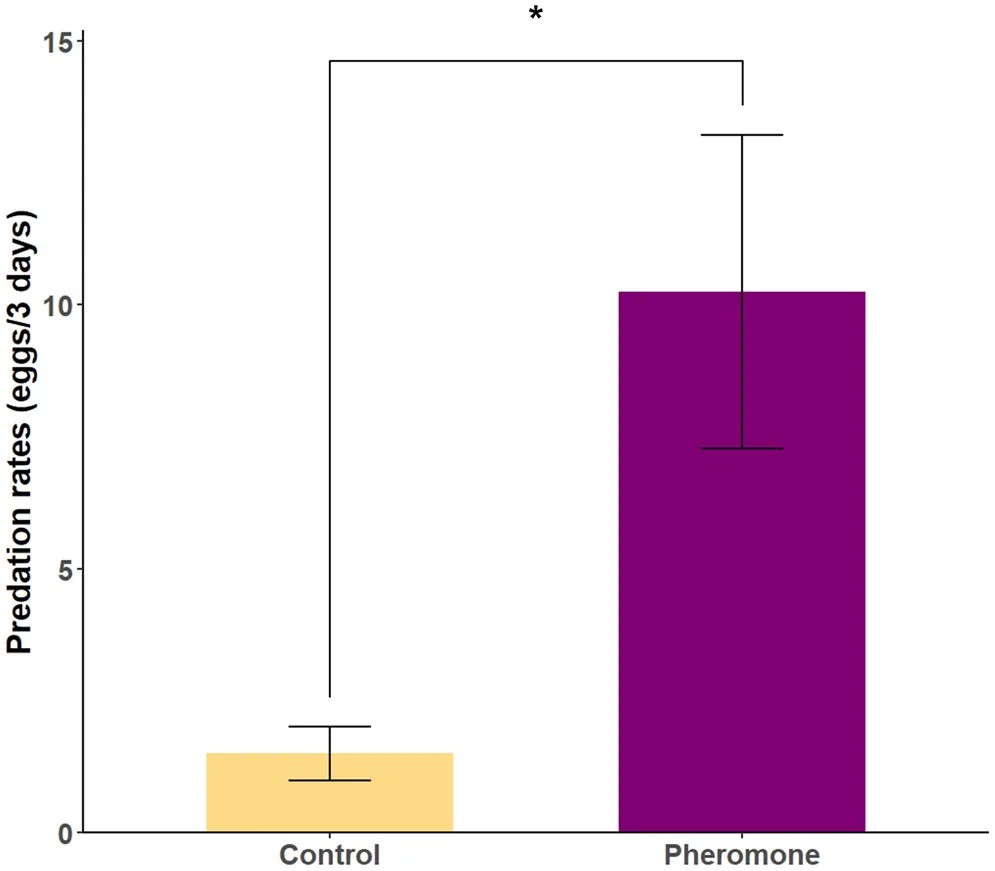
Overall beetle egg predation rates in the control (mean [95% CI]: 1.4 [0.4 to 2.4]) and the pheromone-treated plots (mean [95% CI]: 9.8 [2.7 to 15.9]) over a 2-mo period. Error bars represent standard errors. Asterisk denotes a significant difference between the treatment levels (*P *< 0.05).

Overall egg predation rates over a 2-mo period were higher in the pheromone-treated plots (mean [95% CI]: 9.8 [2.7 to 15.9] eggs per 3 d) compared to the control plots (mean [95% CI]: 1.4 [0.4 to 2.4] eggs per 3 d) (χ² = 11.7; df = 1; *P *< 0.001; Fig. 4).

### Predation of Colorado Potato Beetle Eggs in Greenhouse Assays

**Fig. 5.**
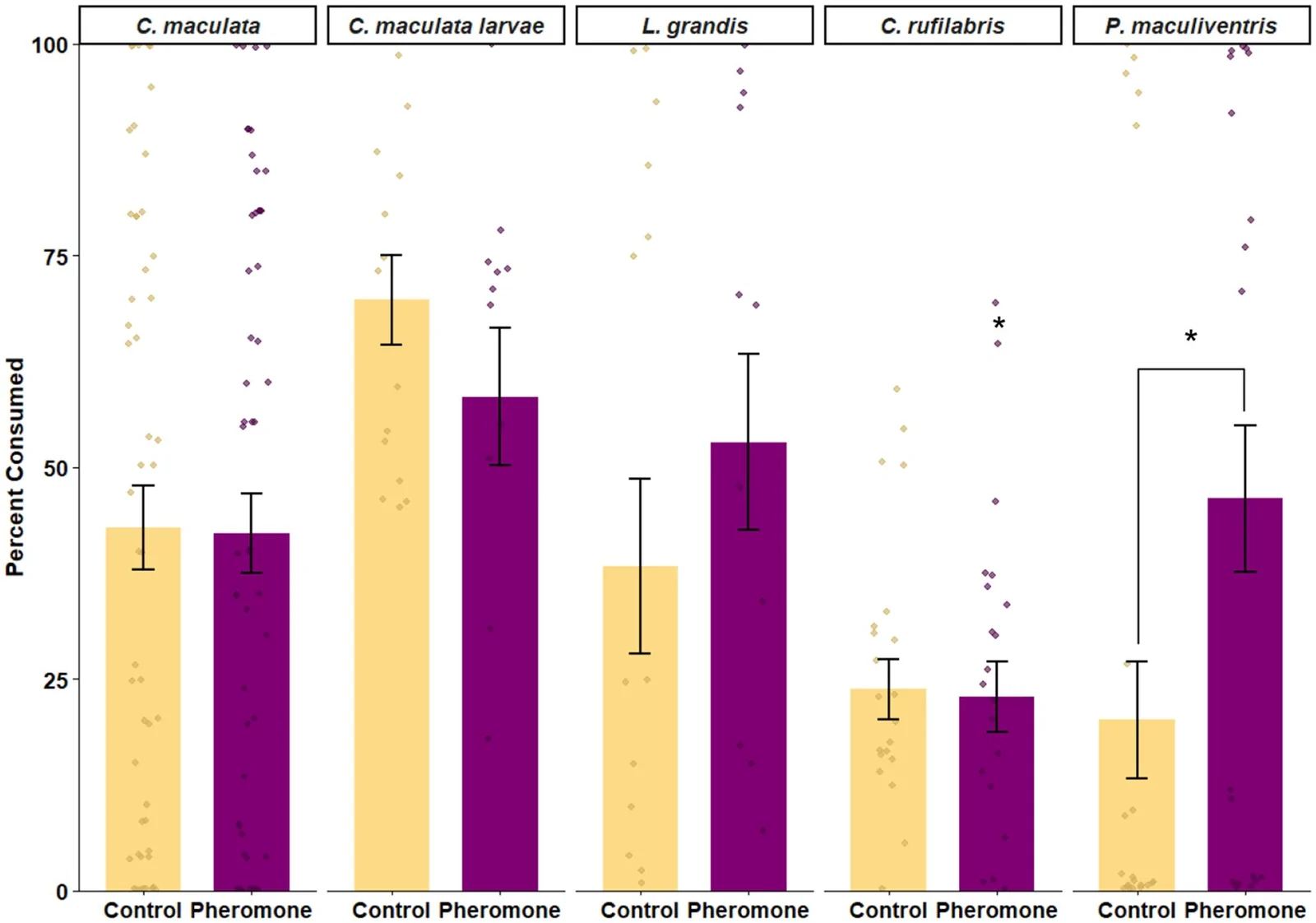
Percent of the Colorado potato beetle eggs consumed by 5 predator species/life stages when exposed to predator pheromone or control volatiles in greenhouse assays. Panels from left to right show *Coleomegilla maculata* adults, *Chrysoperla rufilabris*, *Lebia grandis*, *Podisus maculiventris*, and *Coleomegilla maculata* larvae. Error bars represent standard errors. Asterisks denote significant differences between treatment levels (*P *< 0.05).

There is no significant effect of pheromone treatment on the proportion of eggs consumed by adult *C. maculata* (81.9% vs. 82.9%; *Z *= 0.09; *P *= 0.92; Fig. 5), *C. maculata* larvae (85.5% vs. 68.3%; Z = 0.83; *P = *0.41); *C. rufilabris* (20.7% vs. 13.9%; *Z *= 0.52; *P *= 0.60; Fig. 5), or *L. grandis* (31.1% vs. 66.1%; *Z *= −1.30; *P *= 0.19; Fig. 5).

There is a significant effect of pheromone treatment on the proportion of eggs consumed by *P. maculiventris* (*Z *= −3.69; *P *< 0.001; Fig. 5) with estimated egg consumption increasing from 1.3% in the control treatment to 29.2% in the pheromone treatment.

## Discussion

While the abundance of the target herbivore (the Colorado potato beetle) was reduced by exposure to the synthetic predator pheromone, the abundance, species richness, and diversity of herbivores and natural enemies in the field were not altered throughout the season (Supplementary Figs. S3 and S4). Despite these neutral community responses, predation on beetle eggs was significantly higher in pheromone-treated plots, suggesting that the pheromone may have influenced predator foraging behavior rather than predator abundance. However, predator behavior was not directly measured in this study. Greenhouse assays further revealed that *P. maculiventris* increased its feeding rate in the presence of the pheromone, demonstrating that the compound can enhance consumptive effects for at least one key predator species. In contrast, *C. maculata* lady beetle larvae and adults, lacewing larvae, and *L. grandis* showed no detectable response. Collectively, although predators varied in their feeding responses to the synthetic pheromone, the net outcome positively favored future use of the pheromone in biological control of the Colorado potato beetle.

The lack of community-wide responses by herbivores may reflect differences in how taxa perceive or respond to predator-associated volatiles. Most herbivores in this system, aside from the Colorado potato beetle, are not known to be preyed upon by *P. maculiventris* and therefore may have little incentive to respond directly to its cues. Although previous work demonstrates that predator pheromones can alter plant physiology and quality, both in potatoes exposed to the *P. maculiventris* pheromone (Aflitto and Thaler 2020, Martinez et al. 2025) and in other systems where plants respond to predator-derived cues (Helms et al. 2017), the herbivores in our study did not show detectable changes in a way that suggested response to plant quality. Consistent with this, the Colorado potato beetle responded to predator-pheromone-induced changes in plant quality in previous greenhouse experiments (Martinez et al. 2025), but we found no evidence that other herbivores reacted similarly in our field surveys. Many chewing herbivores adjust behavior rather than population numbers in response to predator cues (Kaplan et al. 2014), though such subtle shifts may have gone undetected as we did not measure behavior. Additionally, herbivores such as leafhoppers and aphids rely heavily on plant nutritional quality and phloem chemistry, traits that may not be strongly influenced by exposure to the predator pheromone (Hewer et al. 2010). Chewing herbivores like flea beetles, despite sharing a feeding mode with Colorado potato beetle larvae, may also differ in how they perceive and respond to predator-associated volatiles (Li et al. 2024).

Aggregation pheromones have been shown to attract adult *P. maculiventris* in field settings, yet such recruitment was not detected in our experiment (Kelly et al. 2014). Predators may vary widely in their responses to the pheromone. Notably, *C. maculata* has been shown to respond to a component of the *P. maculiventris* pheromone under field conditions (Zhu et al. 1999, Kaplan 2012), suggesting capacity for attraction in at least some members of the predator guild. There is evidence that predators can be attracted by HIPVs in field settings; for example, methyl salicylate reliably pulls in diverse predator guilds, showing that single semiochemicals can serve as attractive cues (James 2005, Zhu and Park 2005, Zhang et al. 2006). Additionally, our D-vac and visual sampling methods quantified predator abundance rather than predator activity and may not have detected localized changes in predator behavior within plots. However, predator abundance in our fields remained unchanged across treatments, and predators did not appear to increase their presence in plots with higher Colorado potato beetle larval abundance. This indicates that the pheromone was neither a deterrent nor an attractive cue at the plot scale, nor were any patches of larvae serving as a prey hotspot. Predation rates on Colorado potato beetle eggs changed in the field, prompting us to examine predator feeding behavior in greenhouse assays, where we did observe species-specific responses to the pheromone. *Podisus maculiventris* increased feeding, which may be due to the species’ established use of aggregation pheromones in mate attraction and prey sharing (Sant’Ana et al. 1997). Alternatively, increased egg consumption may reflect a behavioral response to aggregation cues that elevate local predator density, as higher conspecific densities commonly alter per-capita feeding rates through interference or competitive interactions in biological control systems (DeLong and Vasseur 2011). However, this mechanism was not directly tested in our study and warrants future investigation. Although not statistically significant, *L. grandis* exhibited a moderate increase in egg consumption under pheromone exposure, suggesting potential responsiveness among some beetle predators. Not all predators exhibited behavioral responses to the pheromone treatment. *C. maculata* adults did not respond to the predator pheromone. While there is evidence of adult response to an individual component of the pheromone (Zhu et al. 1999, Kaplan 2012) this sensitivity may not translate into attraction or altered feeding behavior in response to a more complex pheromone blend. Similarly, *C. maculata* larvae and lacewings showed no significant differences in feeding rates, suggesting that the pheromone does not universally enhance predation across the natural enemy guild. This lack of response is noteworthy given that these larval predators could be at risk of intraguild predation by adult stink bugs (Mallampalli et al. 2002, Abram et al. 2015). Predator-derived cues have been shown to trigger behavioral and physiological responses in insect prey, though whether such effects consistently scale up to influence population abundance or community structure remains poorly resolved (Hermann and Landis 2017). Such asymmetries highlight the importance of considering functional traits and ecological roles of the larger community when evaluating the potential of semiochemicals as biocontrol tools.

From an applied perspective, our findings demonstrate that predator-derived pheromones can enhance biological control. By increasing predation on Colorado potato beetle eggs, the pheromone has the potential to improve the biocontrol efficiency of key predators, particularly given that Colorado potato beetle feeding damage can reduce potato yield when defoliation exceeds the plants compensatory capacity (Hare 1990, Stieha and Poveda 2015). The low numbers of *P. maculiventris* observed in the field suggest that the increased predation in pheromone-treated plots likely resulted from subtle shifts in predator behavior rather than from increases in predator abundance. This is notable because *P. maculiventris* is difficult to retain in cropping systems and often requires multiple augmentative releases (Biever and Chauvin 1992, Aldrich and Cantelo 1999, Tipping et al. 1999); even a small increase in conspecific attraction, combined with higher per-capita feeding rates, could meaningfully improve its efficacy. Importantly, this enhancement occurred without detectable reductions in the abundance of either beneficial insects or non-target herbivores, indicating that pheromone deployment is unlikely to generate the unintended impacts commonly associated with broad-spectrum insecticides. Although these findings demonstrate the potential of predator pheromones for enhancing biological control, our conclusions are based on a single growing season at one location in a potato production system using a single pheromone release rate. Future studies evaluating multiple environments, cropping systems, years, and pheromone release rates will be important to determine the generality and optimize the application of this approach. Collectively, these outcomes support the integration of predator pheromones into push–pull or attract-and-stimulate strategies to strengthen pest suppression in potato systems.

## Supplementary Material

ieag086_Supplementary_Data
