# Supplementary material for: Evaluating the impact of a synthetic predator pheromone, a novel pest management tool, on target pests and insect communities in potato fields
Source: J Insect Sci. 2026 Aug 19;26(4):ieag086. doi: 10.1093/jisesa/ieag086 (PMC13489717; doi:10.1093/jisesa/ieag086)
Supplement: Supplementary file 1 [file ieag086_supplementary_data.docx]

### Supplementary Materials

**Supplementary Materials Figure S1.** A) Mean abundance, B) Richness, C) Diversity of herbivore taxa in the control and the pheromone-treated plots over a two-month period. Error bars represent standard errors.

**Supplementary Materials Table S1.** Statistical results examining the effects of pheromone treatment on herbivore abundance, richness, and diversity.

| Response variable | Test statistic | df | *p*-value |
| --- | --- | --- | --- |
| *Abundance* | *t* = −0.91 | 139.5 | 0.36 |
| *Richness* | *Z* = −0.22 | --- | 0.83 |
| *Diversity* | *t* = 0.36 | 139.9 | 0.72 |

**Supplementary Materials S2.** A) Mean abundance, B) Richness, C) Diversity of predator taxa in the control and the pheromone-treated plots over a two-month period. Error bars represent standard errors.

**Supplementary Materials Table S2.** Statistical results examining the effects of pheromone treatment on predator abundance, richness, and diversity.

| Response variable | Test statistic | df | *p*-value |
| --- | --- | --- | --- |
| *Abundance* | *t* = −0.12 | 124 | 0.90 |
| *Richness* | *Z* = 0.51 | --- | 0.61 |
| *Diversity* | *t* = 0.84 | 134 | 0.40 |

**Supplementary Materials S3.** Pest abundance shown over the seven-week period. Each line represents the average abundance of herbivores per plot over time. The blue line represents control treatment, and the orange line represents the pheromone treatment.

 **Supplementary Materials S4.** Predator abundance shown over the seven-week period. Each line represents the average abundance of predators per plot over time. The blue line represents control treatment, and the orange line represents the pheromone treatment.
